# Protection of Superconducting Industrial Machinery Using RNN-Based Anomaly Detection for Implementation in Smart Sensor †

**DOI:** 10.3390/s18113933

**Published:** 2018-11-14

**Authors:** Maciej Wielgosz, Andrzej Skoczeń, Ernesto De Matteis

**Affiliations:** 1 Faculty of Computer Science, Electronics and Telecommunications, AGH University of Science and Technology, al. Adama Mickiewicza 30, 30-059 Cracow, Poland; 2 Academic Computer Centre CYFRONET AGH, ul. Nawojki 11, 30-072 Cracow, Poland; 3 Faculty of Physics and Applied Computer Science, AGH University of Science and Technology, al. Adama Mickiewicza 30, 30-059 Cracow, Poland; skoczen@agh.edu.pl; 4 CERN European Organization for Nuclear Research, CH-1211 Geneva 23, Switzerland; ernesto.de.matteis@cern.ch

**Keywords:** anomaly detection, recurrent neural networks, neural networks compression, LHC

## Abstract

Sensing the voltage developed over a superconducting object is very important in order to make superconducting installation safe. An increase in the resistive part of this voltage (quench) can lead to significant deterioration or even to the destruction of the superconducting device. Therefore, detection of anomalies in time series of this voltage is mandatory for reliable operation of superconducting machines. The largest superconducting installation in the world is the main subsystem of the Large Hadron Collider (LHC) accelerator. Therefore a protection system was built around superconducting magnets. Currently, the solutions used in protection equipment at the LHC are based on a set of hand-crafted custom rules. They were proved to work effectively in a range of applications such as quench detection. However, these approaches lack scalability and require laborious manual adjustment of working parameters. The presented work explores the possibility of using the embedded Recurrent Neural Network as a part of a protection device. Such an approach can scale with the number of devices and signals in the system, and potentially can be automatically configured to given superconducting magnet working conditions and available data. In the course of the experiments, it was shown that the model using Gated Recurrent Units (GRU) comprising of two layers with 64 and 32 cells achieves 0.93 accuracy for anomaly/non-anomaly classification, when employing custom data compression scheme. Furthermore, the compression of proposed module was tested, and showed that the memory footprint can be reduced four times with almost no performance loss, making it suitable for hardware implementation.

## 1. Introduction

The benefit of using superconductivity in industrial applications is well understood. However actual application of superconducting devices is still limited by difficulties in the maintenance of cryogenic stability of superconducting cables and coils. In many cases, it is hard to design safe superconducting circuit. A small mechanical rearrangement releases enough energy to initiate local avalanche process (quench) leading to loss of superconducting state and next to overheating of the machine and then even to melting. Therefore superconducting machines require a special protection system.

In general, such a system consists of data producers and processing servers. The producers are electronic devices located close to individual superconducting machines which require protection. This device (producer) is capable of collecting and processing data and generating the activation signal for local actuators. The servers are capable of storing and analyzing data delivered by producers through the network.

The overarching research goal is to improve data processing within the producers and to move a part of the analysis task into them in order to reduce network load. The preliminary results of this research were presented in Ref. [1], which focused on testing the suitability of Recurrent Neural Networks (RNNs) for this application. In this work, the additional aspects for hardware implementation, especially model compression, are explored.

The presented research main contributions are as follows:

- development of a neural algorithm dedicated to detecting anomaly occurring in the voltage time series acquired on the terminals of superconducting machines in electrical circuits,
- design and verification of the complete processing flow,
- introduction of the RNN-based solution for edge computing which paves the way for low-latency and low-throughput hardware implementation of the presented solution,
- development of a system level model suited for future experiments with the adaptive grid-based approach; the software is available online (see Supplementary Material section).

### 1.1. Protection System for Superconducting Machinery

**Figure 1:**
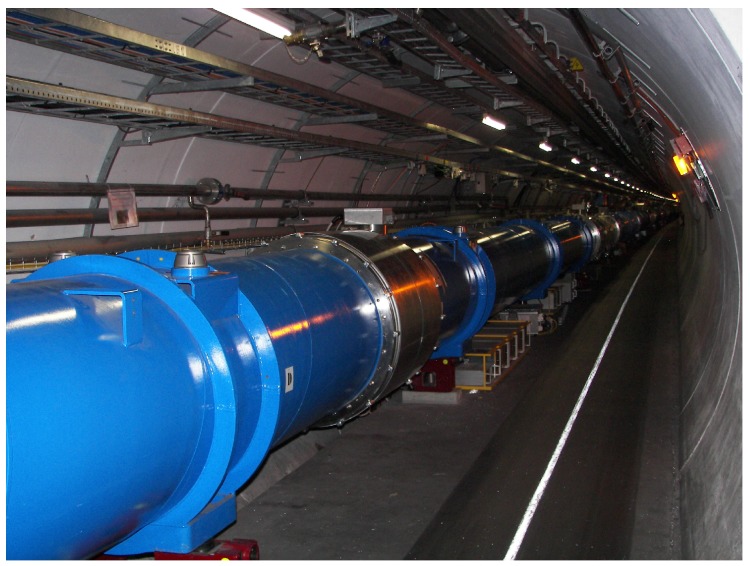
The LHC tunnel. The blue cryostat contains superconducting main dipole magnets. The protection unit is visible on the floor under the magnet (yellow rack). The photo taken by A.S. in 2007.

One of the biggest superconducting systems is installed at the Large Hadron Collider (LHC) accelerator at the European Organization for Nuclear Research (CERN). Despite its scientific purpose, the LHC should be considered as a huge industrial system. The final product of this factory is the total number of particle collisions. During the steady operation, every second, 40,000,000 collisions are performed in three different interaction points. The LHC consists of a chain of superconducting magnets. The chain is located in an underground circular tunnel, 100 m under the Earth’s surface. The Figure 1 presents the view on the magnet’s chain. The perimeter of the tunnel is about 27 km long. Superconducting magnets, responsible for shaping the beam trajectory, are crucial elements of the accelerator that require permanent monitoring. The details of the design of the LHC accelerator are described in Ref. [2].

When the LHC collides particles, even a tiny fraction of energy stored in each proton beam can cause a magnet to leave the superconducting state. Such an occurrence is named a quench, and it can severely damage the magnets in case of machine protection procedures failure. These procedures mainly relay on triggering the power down of the whole accelerator when resistive part of voltage on one superconducting element exceeds a predefined threshold. This study concerns only a protection system of superconducting elements inside the LHC.

A protection system known as Quench Protection System (QPS) has been installed at the LHC since the beginning and it successfully works since ten years of the LHC operation. The detailed description of the existing system can be found in Refs. [3,4]. The protection unit, visible in Figure 1, installed under the magnet performs acquisition, processing and buffering of samples of voltage existing between terminals of the superconducting coil. These units are end-points of a massive distributed system covering the whole length of the LHC. The acquisition is performed using Analog-to-Digital Converter (ADC). The processing relies on filtering and compensation of inductive voltage. The pure resistive voltage is compared with the threshold, and the output of comparator activates actuators and generates trigger signals for other subsystems of the LHC. The actuators are also included to the yellow rack installed under each magnet in the tunnel (see Figure 1). The task of actuators is to inject energy to the objective coil in order to heat it homogeneously.

**Figure 2:**
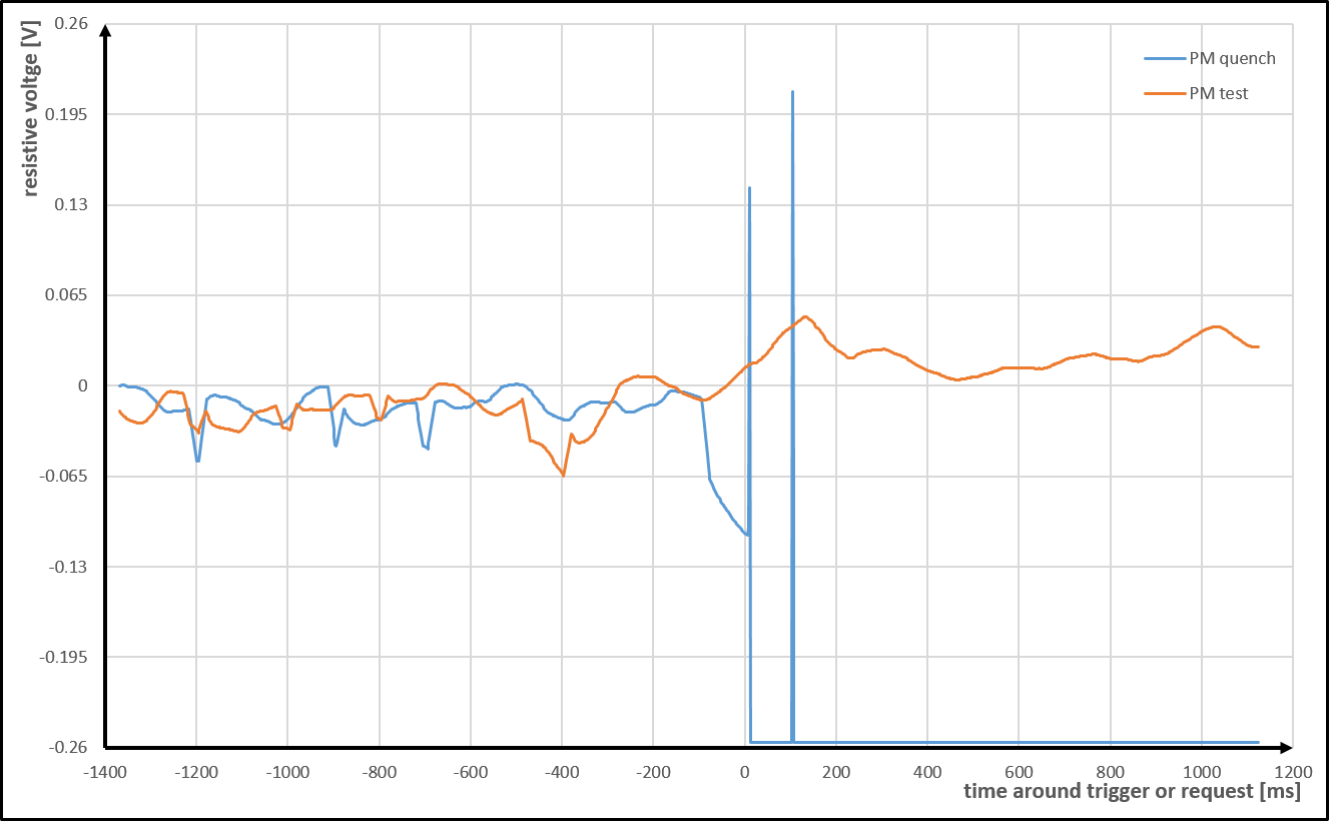
The presentation of a contents of $U_{\mathrm{RES}}$ field of two PM data files for one of the superconducting magnets (with electrical current 600 A). The voltage range of the ADC is from −256 mV to 256 mV. Time 0 ms refers to trigger (request) time stored in the field QUENCHTIME in the PM data files.

The data is continuously buffered in a circular buffer. Some of the samples (both total and resistive voltage) are directly transmitted to the CERN Accelerator Logging Service (CALS) database in the cloud. The CALS serves permanent monitoring of almost every device in the CERN’s accelerator’s complex. The sampling rate for this service is very low, in the best case it is 10 Hz ( 100 ms). The circular buffer consists of two parts. The first part is filled with samples all the time in a circular manner. The second part is filled only in case of triggering or in case of a request sent by an operator. A trigger (or a request) freezes first part of the buffer. Then the whole buffer is transmitted to a cloud and stored in a dedicated database Post Mortem (PM) System. Examples of voltage time series taken from this database are presented in Figure 2. The sampling rate of the PM data is much higher, and in our case, it is 500 Hz ( 2 ms).

**Figure 3:**
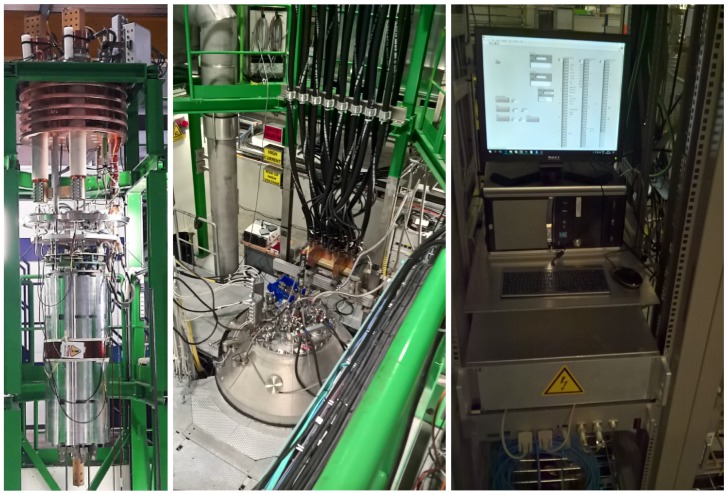
Test facilities of SM18 for testing MQXFS inner triplet quadrupole magnet, including the rack used for the data acquisition and tests (photos provided by E.M.).

The presented protection system underwent many upgrades introduced during breaks of the LHC operation. However, the emergence of new superconducting materials opens a question concerning detection algorithms for application in the future protection system again. Such experiments are conducted in SM18 CERN’s facility (see Figure 3), currently testing the magnets for the High Luminosity LHC phase.

### 1.2. State of the Art

Anomaly and novelty detection methods have been researched over many years [5,6,7] which resulted in the development of many successful algorithms. They may be in general divided into three different categories of density-based, distance-based and parametric methods. In addition to the standard procedures, neural algorithms in a majority of cases employing RNN-based architectures [8,9,10,11] slowly pave the way to the basic set of the anomaly detection procedures.

Training dataset is different for novelty and outlier detection. In novelty detection, it is not contaminated by anomalies—all outliers need to be removed beforehand. On the contrary, the training procedure of the outlier detection model involves incorporating anomalous data into the training dataset. Both flows employ an unsupervised approach as a training procedure, although some of the procedures may be boosted using some external hyper-parameters such as contamination factor, thresholds or max features that are taken into account in the training process.

In this work three different algorithms were used as a baseline for the RNN-based approach proposed by the authors: Elliptic Envelope, Isolation Forest (IF), and One-Class Support Vector Machine (OC-SVM) [12,13,14,15,16,17,18]. Elliptic Envelope belongs to a set of methods with an underlying assumption of known distribution (usually Gaussian) for normal data and all the points distant from the center of the ellipse are considered outliers. The Mahalanobis distance [19] is used as a measure of distance and an indicator that a given data point may be considered as an outlier.

Another useful method, invented in 2008, is the Isolation Forest [20] which performs anomaly detection using the random forest. The underlying concept of this approach is based on an idea of a random selection of features and a random selection of split within the tree nodes between maximum and minimum values of the selected features. A concept of the decision function in IF algorithm defines deviation of an averaged path over a forest of random trees. Random partitioning creates significantly shorter paths for anomalies which results from the fact that outliers are concentrated close to extreme values of features which in turn locates them on the border of the trees.

OC-SVM is usually considered to be a novelty-detection method, and the training data should not contain outliers. It performs well in high-dimensional space where there is no assumption regarding the distribution of the underlying data. However, if the data is well distributed (e.g., Gaussian) the Isolation Forest or Elliptic Envelope may perform better.

The presented methods assume the spatial structure of the data with no temporal relationships. There is a set of methods such as ESD, ARIMA, Holt-Winters [21,22] which take time component into account and have proven to be effective. However, due to their complexity, it is challenging to implement them in very low-latency systems in hardware.

When detecting anomalies in time series, RNNs are both scalable and adaptable which is critical when it comes to design and implementation of complex anomaly detection systems [23,24,25]. RNNs were introduced long ago [26] and have been successfully applied in many domains [8], according to the authors’ knowledge, there are no studies on the performance of compressed RNNs in anomaly detection. Nevertheless, several works on effective quantization and compression of RNNs are available [27,28]. Consequently, decision was made to explore the feasibility and performance of several compression techniques of RNNs in low-latency anomaly detection domain of LHC machinery monitoring. Furthermore, the adopted approach addresses both data and coefficients quantization with in-depth analysis of correlation of employing different techniques.

## 2. Materials and Methods

### 2.1. Quantization Algorithm

#### 2.1.1. Previous Work

In the authors’ previous works concerning superconducting magnets monitoring Root-Mean-Square Error (RMSE) [24] and both *static* [23] and *adaptive* data quantization [1,25] approaches were used. Based on experiments conducted and described therein, a conclusion can be drawn that RNNs can be used to model magnets behavior and detect anomalous occurrences.

**Figure 4:**
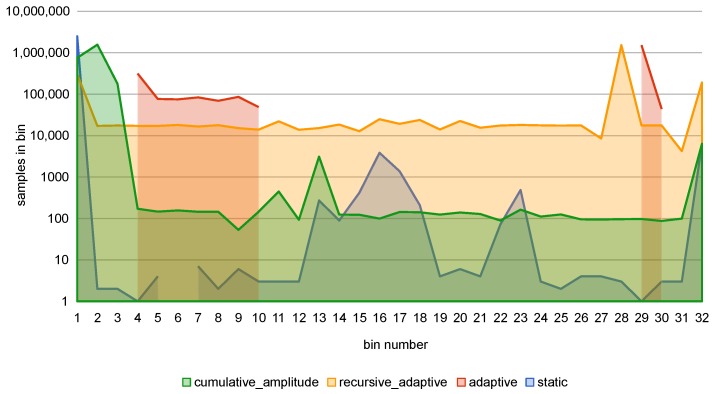
Samples per bin for PM dataset $U_{\mathrm{RES}}$ channel (m=32). Note the logarithmic scale.

The initially introduced RMSE approach had several drawbacks, the main of which was a necessity to select an arbitrary detection threshold. In Ref. [23], the *static* quantization was used, mapping the input data into a set of *m* equal-width bins. This method, however, resulted in sub-par results, stemming from the uneven distribution of the samples in the bins, up to the point where nearly all samples occupied only one or two bins (see *static* samples counts in Figure 4).

**Figure 5:**
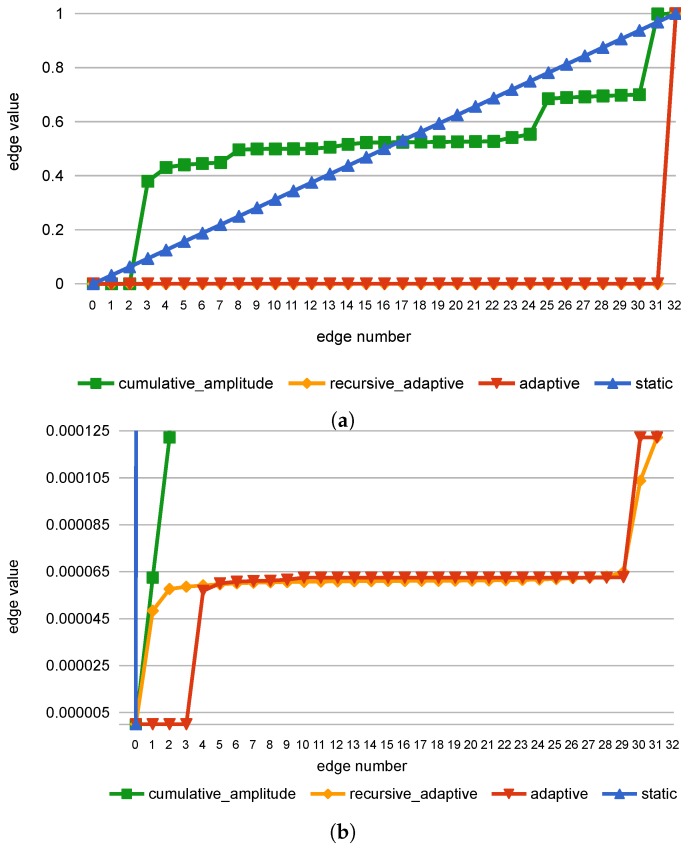
Full (**a**) and zoomed-in (**b**) bin edges for PM dataset $U_{\mathrm{RES}}$ channel (m=32). Please note that *adaptive* quantization algorithm effectively yields only 10 bins, since some edges values occur multiple times.

In Ref. [25], an approach based on *adaptive* data quantization and automatic thresholds selection was introduced. *Adaptive* data quantization resulted in much better use of bins and consequently significantly improved the accuracy results. Its principle of operation is mapping the input space to a fixed number of categories (bins) in such a way, that all categories have (ideally) the same samples cardinality (see Appendix A.1 for more details). Resulting bins widths are uneven, explicitly adjusted to each of the input signal channels (see *adaptive* samples counts in Figure 4 and Figure 5 to compare bin edges generated with various approaches).

#### 2.1.2. Other Quantization Approaches

The drawback of *adaptive* algorithm is that it can effectively generate fewer bins than requested when some values occur in the dataset in significant numbers (see Figure 5). To mitigate this effect, which became apparent when working with PM data, a modification of *adaptive* algorithm, called *recursive_adaptive*, was introduced. The m+1 initially found edges are treated as candidates, and if duplicates are detected, the recursive process is started. At first, the duplicated edges are added to the final edges list, and all repeating values are removed from the dataset. Then, the remaining data is used as an entry point to find m+1–*number of final edges* new edge candidates. The process is repeated until there are no duplicates in candidate edges or there is no more data left in the dataset. As a result of this process, the bins are more evenly used.

An alternative edge-finding algorithm, called *cumulative_amplitude*, is based on the idea of equalizing the sum of the samples amplitudes in each bin. As in *adaptive* algorithm, before edges selection, the samples are normalized and sorted. Then, the threshold value is computed as a sum of amplitudes of samples left in the dataset divided by the required edges number (see Appendix A.2 for equations). Contrary to the *adaptive* approach of determining the edges based on the samples count, in *cumulative_amplitude* the edge value is chosen when the sum of samples’ amplitudes crosses this threshold. As a result, the maximal values are not grouped with smaller ones. It may, however, level the differences between smaller values, that may contain crucial information. In the implementation, the concept described above was modified to also use recursive duplication removal, with the threshold value determined anew for each recursion level.

### 2.2. Implementation Overview

**Figure 6:**
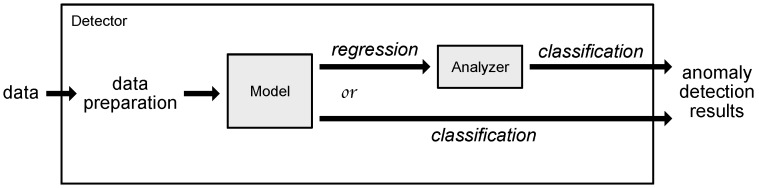
High-level system architecture. © 2018 IEEE. Reprinted, with permission, from Wielgosz, M.; Skoczeń, A.; Wiatr, K. Looking for a Correct Solution of Anomaly Detection in the LHC Machine Protection System. 2018 International Conference on Signals and Electronic Systems (ICSES), 2018, pp. 257–262 [1].

The presented anomaly detection system was created in Python, using Keras [29] library, with both Theano [30] and Tensorflow [31] backends depending on availability. The reference methods were implemented using scikit-learn [32] library. It is prepared to work with normalized data, with all the available data (both training and testing) used during the normalization process. The focal system modules and data flow can be seen in Figure 6.

The number of input categories (in_grid), the bins’ edges calculation algorithm (in_algorithm), the history window length (look_back), the model and its hyper-parameters used during the anomaly detection process and other options are specified in the configuration file. The particular setup is also saved while results are reported, ensuring the particular test environment can be recreated even if configuration included several possible values. Each of the input channels is quantized using the same grid/algorithm combination.

The model is an abstraction layer over the actual classifier. Currently implemented models include Random (for baseline testing), Elliptic Envelope, Isolation Forest, OC-SVM, Long Short-Term Memory (LSTM) and Gated Recurrent Unit (GRU).

Depending on the system configuration (Figure 6), some models can be used for either classification or regression. In the regression mode, the model is trained on data without anomalies and yields the output that needs to be further processed by the analyzer to obtain anomaly detection results.

In the classification mode, used in the experiments presented in this paper, the model is trained using data containing anomalies (except for models belonging to the novelty detection category). Instead of trying to predict the next (quantized) value, it directly classifies the sample as either anomaly or not.

The models can be roughly divided into the ones working with either spatial (Elliptic Envelope, Isolation Forest, OC-SVM) or temporal data (LSTM, GRU). Relevant data preprocessing and structuring, as well as model training and testing, is coordinated by one of the possible detectors. The model/detector combination used in particular setup is defined in configuration file. This option ensures the system extensibility since the detector does not need to know about all possible models in advance.

**Figure 7:**
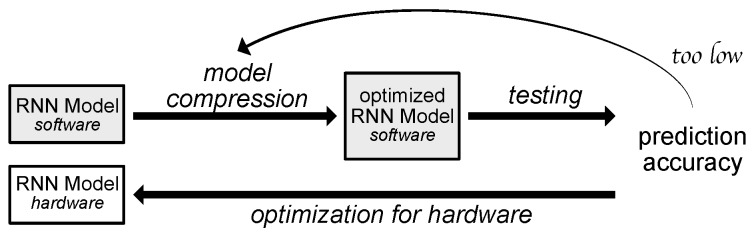
Design flow for hardware implementation. © 2018 IEEE. Reprinted, with permission, from Wielgosz, M.; Skoczeń, A.; Wiatr, K. Looking for a Correct Solution of Anomaly Detection in the LHC Machine Protection System. 2018 International Conference on Signals and Electronic Systems (ICSES), 2018, pp. 257–262 [1].

**Figure 8:**
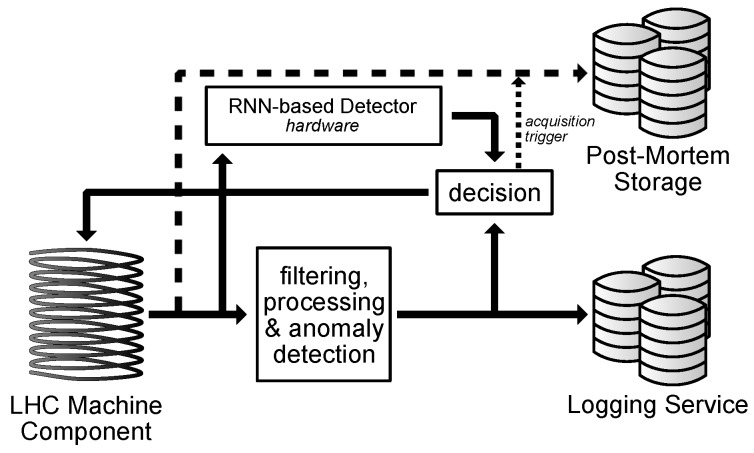
Proposed system. © 2018 IEEE. Reprinted, with permission, from Wielgosz, M.; Skoczeń, A.; Wiatr, K. Looking for a Correct Solution of Anomaly Detection in the LHC Machine Protection System. 2018 International Conference on Signals and Electronic Systems (ICSES), 2018, pp. 257–262 [1].

Currently, the experiments are carried out using the software implementation of the system. The target system, however, will need to be implemented in hardware to ensure it complies with latency requirements. To fit the Neural Network (NN) model onto the Field-Programmable Gate Array (FPGA) or Application-Specific Integrated Circuit (ASIC) board, it needs to be compressed while retaining the high accuracy (Figure 7). The ready module can potentially be used as a stand-alone detector or in conjunction with the currently used system (Figure 8).

### 2.3. Model Complexity Reduction

Deep Learning models have a range of features which render them superior to other similar Machine Learning models. However, they usually have high memory footprint as well as require substantial processing power [33]. Computing requirements are especially crucial when it comes to embedded implementation of Deep Learning models in edge processing nodes like in the case of the system described in this paper. Fortunately, there are multiple ways to mitigate these issues and preserve all the benefits of the models, for example by using techniques such as pruning and quantization [28].

During a quantization, a floating-point number *x* from a quasi-continuous space of IEEE-754 notation is mapped to fixed-point value *q*, represented using total bits. The total is conventionally equal to 8 or 16, which are bit-widths supported by GPUs and the latest embedded processors. For FPGA and ASIC it is, however, possible to use an arbitrary number of bits. The quantization is done separately for each layer’s weights W.

#### 2.3.1. Linear Quantization

The *linear* quantization used during experiments can be described by the following equation:

(1) $$q=s\;\cdot \;\mathrm{clip}\left(\left\lfloor \frac{x}{s}+\frac{1}{2}\right\rfloor ,1-2^{\mathrm{total}-1},2^{\mathrm{total}-1}-1\right),$$

where scaling factor:

(2) $$s=\frac{1}{2^{\mathrm{total}-1-\left\lceil \log_{2}\max(W)\right\rceil }}$$

and clipping function:

(3) $$\mathrm{clip}\left(a,min,max\right)=\left\{\begin{array}{cc} min & \mathrm{if}\;a<min,\\ a & \mathrm{if}\;min\leq a\leq max,\\ max & \mathrm{otherwise}. \end{array}\right.$$

#### 2.3.2. MinMax Quantization

The *minmax* quantization used during experiments can be described by the following equation:

(4) $$q=s\;\cdot \;\left\lfloor \frac{x-\min(W)}{s}+\frac{1}{2}\right\rfloor +\min\left(W\right),$$

where scaling factor:

(5) $$s=\frac{\max(W)-\min(W)}{2^{\mathrm{total}}-1}.$$

Also tested was *log_minmax* quantization, where:

(6) $$q=\mathrm{sign}\left(x\right)\;\cdot \;e^{\mathrm{minmax}(\ln\left|x\right|)}$$

#### 2.3.3. Hyperbolic Tangent Quantization

The *tanh* quantization used during experiments can be described by the following equation:

(7) $$q=\mathrm{arctanh}\left(s\;\cdot \;\left\lfloor \frac{\tanh(x)+1}{s}+\frac{1}{2}\right\rfloor -1\right),$$

where scaling factor:

(8) $$s=\frac{2}{2^{\mathrm{total}}-1}.$$

The main idea behind coefficients quantization is using a dynamic range of the available number representation to its fullest, and meet the requirements of the hardware platform to be used for deploying the system at the same time. In our implementation, the so-called dynamic fixed-point notation was used. It allows emulating fixed-point number representation using floating-point container. It is worth noting that most of edge computing platforms require linear quantization due to the fixed, a priori defined size of internal registers and arithmetic processing elements. FPGA and custom-designed ASIC which this work is targeting have no such limitation.

## 3. Results

A series of experiments were conducted to practically examine performance of the proposed methods. Different configurations of the module setup were used in order to expose impact of different parameters of the proposed algorithm on the overall performance of the anomaly detection system. Furthermore, the performance of the proposed solution was compared with a range of state-of-the-art algorithms.

### 3.1. Dataset

The dataset used in the experiments contained 2500 series retrieved from PM database, with 64-16-20 training-validation-testing split. 2415 of those series had a length of 1248 samples, while the remaining 85 series had a length of 1368 samples. For each of the series, the four input channels were available:

$U_{\mathrm{DIFF}}$—total voltage measured between terminals of superconducting coil,
$U_{\mathrm{RES}}$—resistive voltage extracted from the total voltage $U_{\mathrm{DIFF}}$ using the electric current $I_{\mathrm{DCCT}}$,
$I_{\mathrm{DCCT}}$—current flowing through superconducting coil measured using Hall sensor, and
$I_{\mathrm{DIDT}}$—time derivative of the electric current $I_{\mathrm{DIDT}}$ calculated numerically.

Anomalies were marked based on the value of QUENCHTIME field found in PM data, with each anomaly starting at the indicated point and continuing until the end of a series. As such, the data can be considered to be weakly labelled. 874 training and 225 testing series contained anomalies and over 26% of samples in the dataset were marked. Over 84% of the anomalies had a length of 750 samples, over 7%—566, and over 4%—1320. The length of the remaining anomalies varied between 214 and 908 samples.

**Figure 9:**
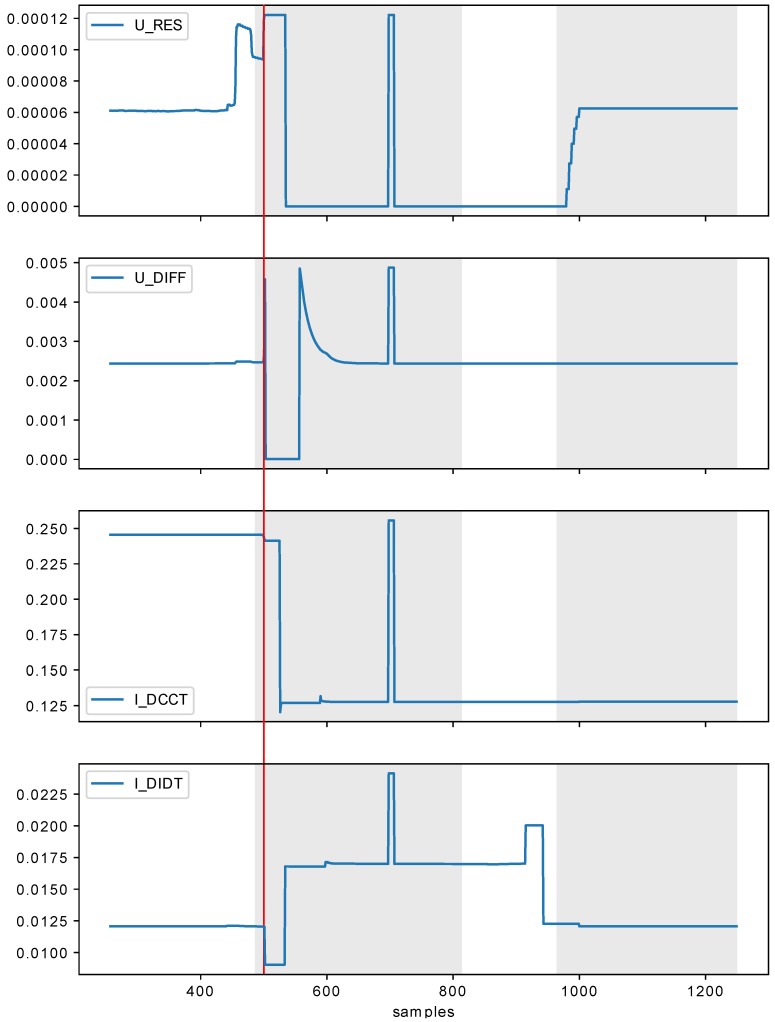
Example single series results visualization (in_grid=32, in_algorithm=adaptive, look_back=256). Red line across all subplots marks the QUENCHTIME and gray spans indicate the anomalies found by the system.

**Figure 10:**
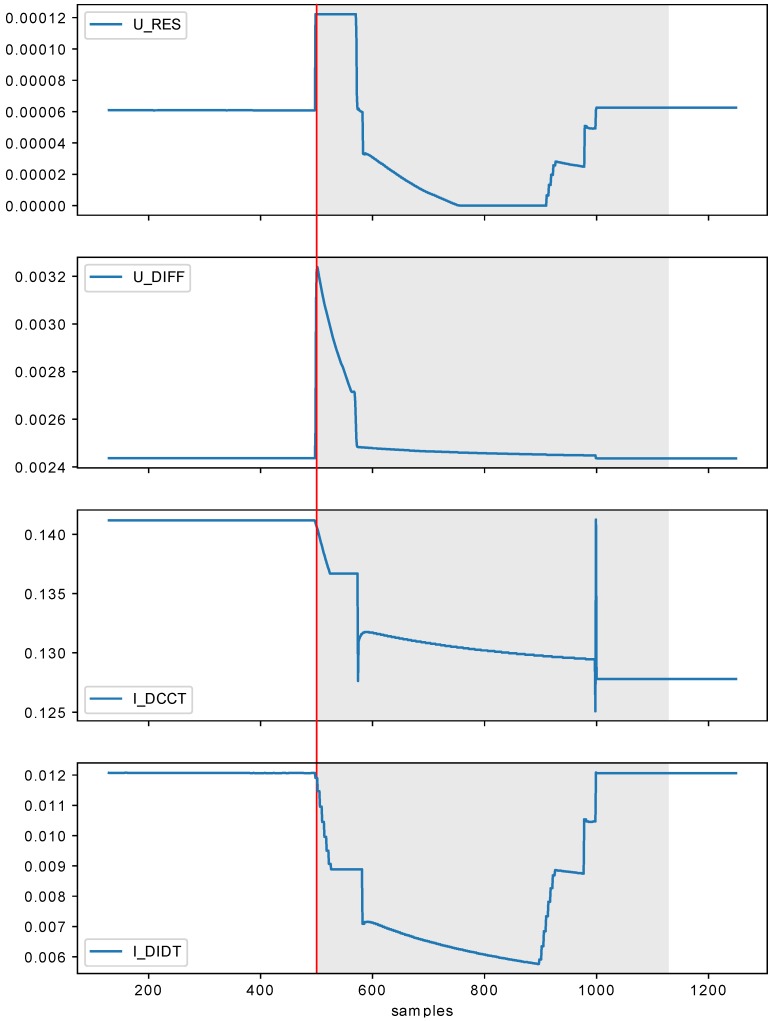
Example single series results visualization (in_grid=32, in_algorithm=recursive_adaptive, look_back=128). Red line across all subplots marks the QUENCHTIME and gray spans indicate the anomalies found by the system.

Before the start of experiments, the data was normalized. The example data series (and results) visualizations can be seen in Figure 9 and Figure 10. Even in just those two figures, it can be seen that the quenches vary in shape and it is really difficult to find apparent similarities just by visual examination. This makes tasks of data labeling and manual verification of the detection results not feasible without heavy experts involvement.

### 3.2. Quality Measures

During the experiments two main quality measures were used: F-measure and accuracy. While F-measure is better suited to evaluate the results of anomaly detection, in case of PM data the relative lack of imbalance between anomalous and normal samples (especially factoring in the required history length) makes the accuracy also a viable metric.

Additionally, the NN models quantization results are usually measured in terms of accuracy, so its usage allows to relate our results with others found in literature. For example, the drop in accuracy resulting from quantization should be no higher than one percentage point [34].

An accuracy can be defined as:

(9) $$\mathrm{accuracy}=\frac{\mathrm{tp}+\mathrm{tn}}{\mathrm{tp}+\mathrm{tn}+\mathrm{fp}+\mathrm{fn}},$$

where:

- tp—true positive—item correctly classified as an anomaly,
- tn—true negative—item correctly classified as a part of normal operation,
- fp—false positive—item incorrectly classified as an anomaly,
- fn—false negative—item incorrectly classified as a part of normal operation.

An F-measure is calculated using two helper metrics, a recall (10), also called sensitivity, and a precision, also called specificity (11):

(10) $$\mathrm{recall}=\frac{\mathrm{tp}}{\mathrm{tp}+\mathrm{fn}},$$

(11) $$\mathrm{precision}=\frac{\mathrm{tp}}{\mathrm{tp}+\mathrm{fp}}.$$

The β parameter controls the recall importance in relevance to the precision when calculating an F-measure:

(12) $$F_{\beta }=\left(1+\beta^{2}\right)\;\cdot \;\frac{\mathrm{recall}\;\cdot \;\mathrm{precision}}{\mathrm{recall}+\beta^{2}\;\cdot \;\mathrm{precision}}.$$

During the experiments two β values were used, 1 and 2, to show the impact of the recall on the final score. Recall as a quality assessment measure reflect an ability of an algorithm to find all entities. On the other hand precision, describes several found entities were correctly classifier. Those measures have to some extent opposite effect on each other. This means that raise of precision usually leads to a drop of recall and vice-versa.

The Receiver Operating Characteristic (ROC) curve is a graph used to analyze the operation of the binary classifiers as the one presented in this work. It shows the performance of the model taking into account all classification thresholds. True Positive Rate (recall) is plotted as a function of False Positive Rate (1−precision). When a classification threshold is lowered, the classifier tends to classify more input data items as positive, which leads to an increase of both fp and tp.

To derive quantitative conclusions from ROC curve Area Under Curve (AUC) may be employed. It measures the whole two-dimensional area under the ROC curve. It may be considered as an integral operation performed from points (0,0) to (1,1) on a ROC graph. AUC values fall into a range between 0 and 1. A model whose predictions are 100% wrong has an AUC of 0.0, the one which works perfectly hasAUC of 1.0.

### 3.3. History Length and Data Quantization

The initial experiments attempted to determine the impact of history length (look_back) and quantization levels (in_grid) on RNN models performance. Models were trained on full dataset and four channels for 7 epochs, with batch size equal to 16,384.

**Figure 11:**
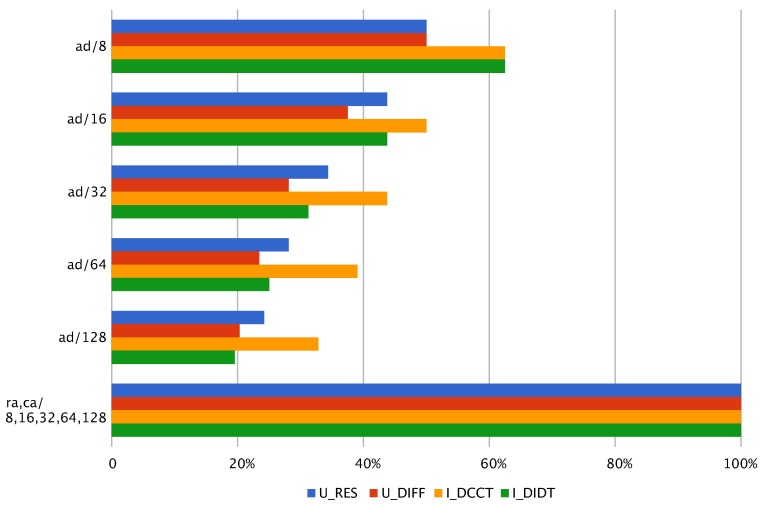
Grid use for various in_grid values and in_algorithm. ad—*adaptive*, ra—*recursive_adaptive*, ca—*cumulative_amplitude*.

Use of dynamic range of the data representation is one of the most important indicators of the quantization algorithm effectiveness since it affects the potential information loss due to the lack of a proper representation capacity, i.e., ‘wasting’ resources on empty bins, while other bins contain ‘too many’ of the values or the number of bins could be reduced altogether. Figure 11 shows that recursive and cumulative adaptive approaches provide full grid use in contrast to adaptive quantization method which exhibits significant grid underuse.

**Figure 12:**
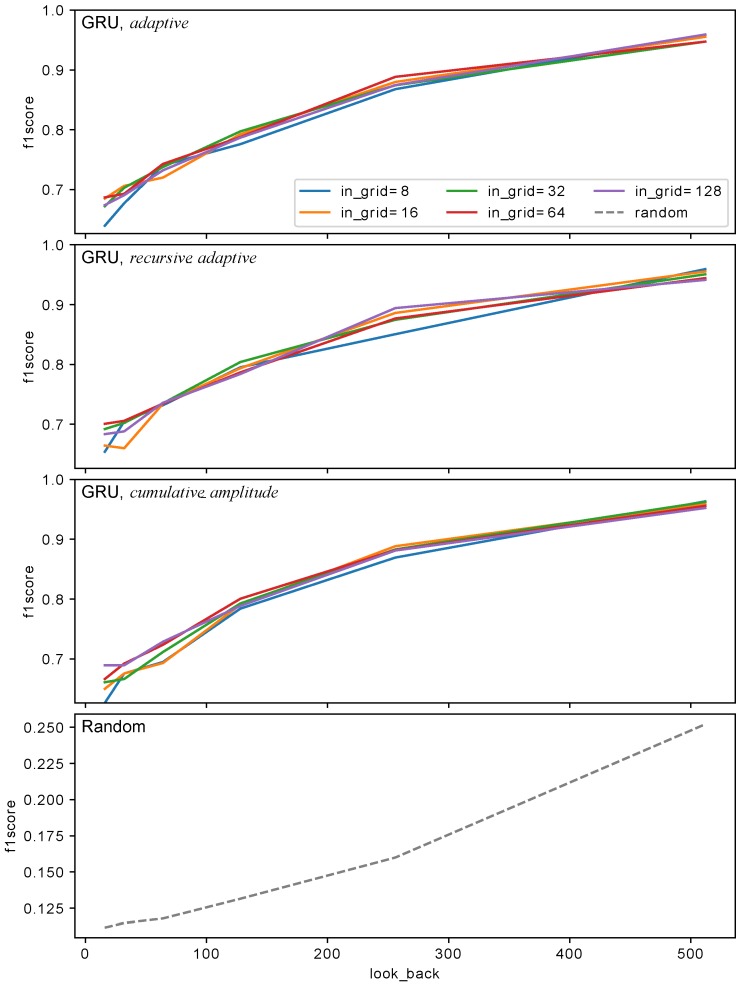
$F_{1}$ score as a function of look_back for several in_grid and in_algorithm values. Dashed line shows Random baseline model performance for the same look_back.

**Figure 13:**
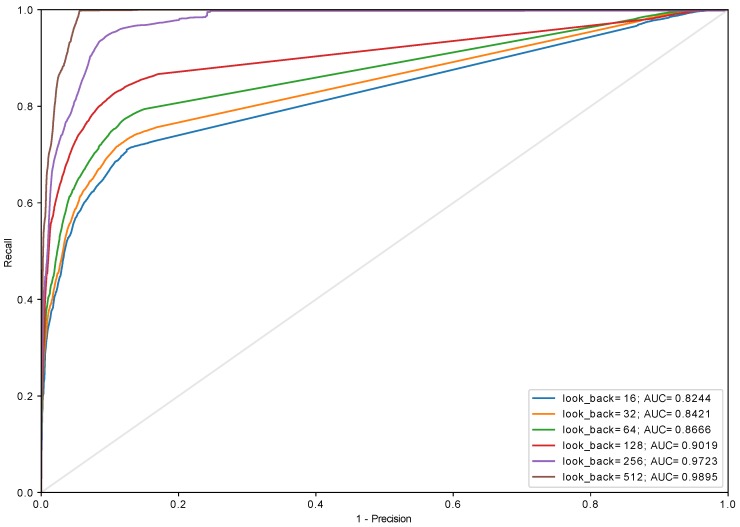
The ROC curve for algorithm *adaptive* (in_algorithm=adaptive, in_grid=32, GRU (two layers, 64 and 32 cells) + Dense).

**Figure 14:**
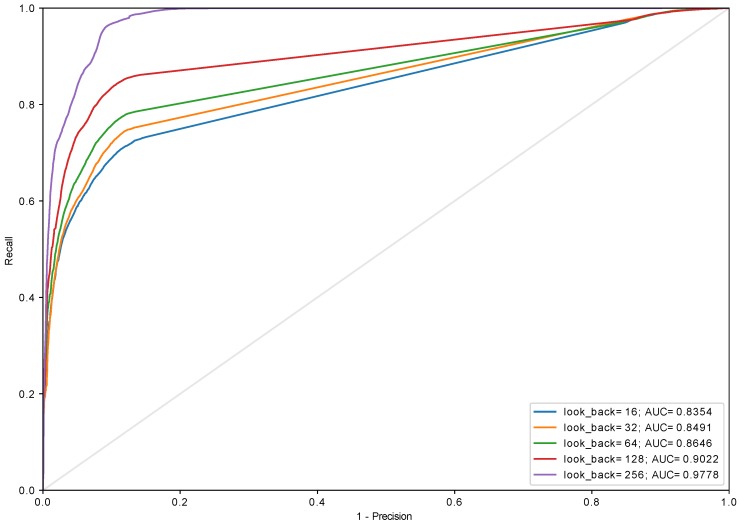
The ROC curve for algorithm *recursive_adaptive* (in_algorithm=recursive_adaptive, in_grid=32, GRU (two layers, 64 and 32 cells) + Dense).

**Figure 15:**
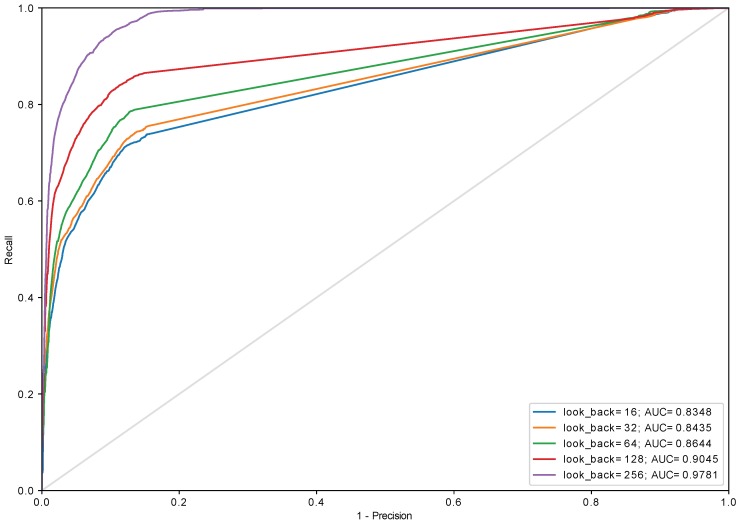
The ROC curve for algorithm *cumulative_amplitude* (in_algorithm=cumulative_amplitude, in_grid=32, GRU (two layers, 64 and 32 cells) + Dense).

Experiments with different sizes of in_grid were conducted and it turned out that this parameter has a very little impact on the performance of the model (see Figure 12). Aside from in_grid=8, which universally performs the worst regardless of the used algorithm, other values yield similar results starting with look_back=128. This allows reducing the size of the input (in_grid) to 32 which can be encoded using 5 bits. The biggest impact on the performance has look_back which was presented in Figure 13, Figure 14 and Figure 15 and Table 1. The models with look_back of 512 reach AUC close to 0.98 and significantly outperform the models with look_back of 16. It also can be seen that in current tests only setups with look_back=256 and look_back=512 were capable of reaching the recall=1, ensuring all anomalies were found, while retaining high precision. The avoidance of false negatives is crucial in this use case, since quench after-effects, resulting in the equipment destruction, can be both dangerous and extremely costly.

It is also worth to keep in mind that the developed model is supposed to work in highly demanding environment where response latency is a critical factor which decides how much time other sections of the global protection system have to execute their procedures. Thus the work needs to be done towards reducing discrepancy in AUC value between setups of different look_back. For instance, AUC for look_back = 64 is 0.87 and for look_back = 256 equals to 0.97 (see Figure 13).

The comparative tests were run using full training set (samples_percentage=1). The goal was to study the behavior of the RNN-based methods and other classic anomaly detection methods with respect to the quantization algorithm. Most of the tests were run using single $U_{\mathrm{RES}}$ input channel (Table 2), with additional experiments using four input channels run for RNN-based methods (Table 3).

As a baseline, the Random model was used. It generates predictions by respecting the training set’s class distribution (“stratified” strategy). Since it ignores the input data entirely, its results do not depend on the in_algorithm choice. This model performance turned out to be at the level of 0.6334.

For the other models’ tests, based on the previous experiments, the values of look_back=256 and in_grid=32 were selected as providing a good tradeoff between resources consumption and the results quality. For the spatial methods, the history vector was flattened. The amount of contamination (the proportion of outliers in the data set) was calculated based on the whole training set and passed to the methods. For the Elliptic Envelope, all points were included in the support of the raw Minimum Covariance Determinant (MCD) estimate. The OC-SVM was tested with both 332 Radial Basis Function (RBF) and linear kernel, with RBF kernel coefficient (γ) equal to 0.1 and an upper bound on the fraction of training errors and a lower bound of the fraction of support vectors (ν) equal to 0.95∗contamination+0.05. Each of the RNNs had the same testing architecture (two layers, with 64 cells in the first one and 32 cells in second, followed by fully connected layer) and was trained for seven epochs.

It is worth noting that the presented methods such as OC-SVM, Isolation Forest, or Elliptic Envelope are more sensitive to the data distribution and perform well for specific kind of underlying data (i.e., specific distribution or feature extraction which can bring the original data to this distribution before the methods are applied). In the case of the presented CERN data, the distribution is not always stable (varies across setups and magnets). It is also worth noting that the distribution as such does not tell much about temporal aspects of the analyzed data. It may happen that the signals of the same distribution have different temporal shape. This is even more pronounced for more temporarily complex signals (see Figure 9 and Figure 10).

### 3.4. Coefficients Quantization

Table 4 shows results of coefficients quantization for the neural models from Table 2 and Table 3 using several different methods. For all methods, the quantization above the ten bits yields results nearly identical to original. A significant drop in accuracy is observed below 8 bits of representation. It may be noted (Table 3) that for lower number of bits the performance oscillates between ≈0.7 and ≈0.3 which means classifying all the features as one category.

## 4. Discussion and Conclusions

The protection system for superconducting machinery existing at the LHC is a vast distributed system installed around the whole circular tunnel. It consists of many individual units connected with a dedicated network. The approach used to design protection units is hard to scale and requires laborious manual adjustment of working parameters. The presented work explores the possibility of using the RNN to build a protection device of a new generation. The idea is to perform on-line analysis inside local protection unit using data acquired with a much higher sampling rate without sending such a massive amount of data to the cloud.

One of the main advantages of the proposed methodology is the simplicity of the parameters setting and adjustment through a complete workflow. Only the model architecture and data quantization levels need to be selected, and even those can be automatically optimized. Additionally, since the solution is based on NNs, it can be extended (scaled) to use more sensors (or data streams) or even incorporate text tags (present in many cases in historical CERN superconducting magnets data), while keeping the overall architectural design the same, even for various types of magnets. It is also possible to fine-tune or retrain RNN-based modules when data has changed (e.g., underlying architecture was modified or aged) when in the traditional approach it would require reconsideration and restructuring of the existing solution. This architectural uniformity makes it a good candidate for implementation in a distributed edge-computing cluster of sensors, trained in an end-to-end fashion. Such a holistic approach can significantly reduce overall resources consumption, latency, and throughput.

The conducted experiments showed that using large look_back significantly boost the performance of the model, while the number of quantization levels (in_grid) as low as 32 is sufficient for the task. The framework demonstrated to be capable of achieving 93% of testing accuracy for GRU (two layers, 64 and 32 cells). The accuracy results are affected by the weak labeling of the data, e.g., sometimes the system labels as anomalous samples occurring for a bit before QUENCHTIME marker. Such results lower the accuracy, while in fact being the desired outcome. The proposed system also often creates a ‘gap’ in the anomaly, at which point the system shutdown signal would already be sent (such example can be seen in Figure 9). Considering that, the availability of the data manually labeled by experts could improve the system performance.

The coefficients quantization level should also be considered a meta-parameter of the model optimization. Experiments showed that the selection of any value equal to or above 8 bits does not lead to noticeable performance degradation. Careful choice of the quantization level may allow reducing memory footprint even more; however, it must be noted that below 8 bits the accuracy of the model oscillates.

Overall, due to the relatively small size of the neural models and the possibility of significantly reducing their memory footprint (4×) with a minimal performance loss, the presented model is a good candidate for hardware implementation in FPGA or ASIC.

## Figures and Tables

**Table 1:** The parameters of NN built with GRU cells for three different algorithms (two layers, 64 and 32 cells + Dense, in_grid=32).

In_Algorithm	Look_Back	Accuracy	$F_{1}$ Score	$F_{2}$ Score
*adaptive*	16	0.8462	0.6722	0.6167
	32	0.8506	0.7031	0.6687
	64	0.8611	0.7376	0.7124
	128	0.8838	0.7973	0.7835
	256	0.9162	0.8743	0.8796
	512	0.9543	0.9474	0.9522
*recursive_adaptive*	16	0.8507	0.6920	0.6481
	32	0.8543	0.7022	0.6561
	64	0.8652	0.7350	0.6928
	128	0.8868	0.8040	0.7939
	256	0.9172	0.8746	0.8749
	512	0.9571	0.9506	0.9560
*cumulative_amplitude*	16	0.8436	0.6609	0.5999
	32	0.8473	0.6664	0.5968
	64	0.8562	0.7115	0.6620
	128	0.8853	0.7927	0.7622
	256	0.9231	0.8830	0.8805
	512	0.9669	0.9625	0.9779

**Table 2:** Testing accuracy (20% of dataset). All models were run with in_grid=32 and look_back=256, using single input channel ($U_{\mathrm{RES}}$), NNs were trained for 7 epochs. The best result is marked in bold.

Model	In_Algorithm		
	*Adaptive*	*Recursive_Adaptive*	*Cumulative_Amplitude*
Random (stratified)	0.6334	0.6334	0.6334
Elliptic Envelope	0.6700	0.7775	0.6700
Isolation Forest	0.7947	0.7596	0.8094
OC-SVM (RBF kernel)	0.3300	0.8232	0.3300
OC-SVM (linear kernel)	0.2959	0.7881	0.2528
GRU (two layers, 64 and 32 cells)	0.8928	**0.9005**	0.8842
LSTM (two layers, 64 and 32 cells)	0.8271	0.8552	0.7402

**Table 3:** Testing accuracy (20% of dataset). Models were run with in_grid=32 and look_back=256, using four input channels ($U_{\mathrm{RES}}$, $U_{\mathrm{DIFF}}$, $I_{\mathrm{DCCT}}$, $I_{\mathrm{DIDT}}$), NNs were trained for 7 epochs. The best result is marked in bold.

Model	In_Algorithm		
	*Adaptive*	*Recursive_Adaptive*	*Cumulative_Amplitude*
GRU (two layers, 64 and 32 cells)	0.9235	**0.9300**	0.8842
LSTM (two layers, 64 and 32 cells)	0.9194	0.9092	0.9023

**Table 4:** Coefficients Quantization Results for GRU (two layers, 64 and 32 cells) + Dense, trained on four input channels. Accuracy as a function of bit-width.

Bits	Method	In_Algorithm		
		*Adaptive*	*Recursive_Adaptive*	*Cumulative_Amplitude*
**Original Model**		**0.9235**	**0.9300**	**0.8842**
10	*linear*	0.9236	0.9287	0.8841
	*minmax*	0.9233	0.9300	0.8841
	*log_minmax*	0.9235	0.9298	0.8842
	*tanh*	0.9232	0.9283	0.9232
9	*linear*	0.9236	0.9279	0.8838
	*minmax*	0.9237	0.9295	0.8842
	*log_minmax*	0.9231	0.9293	0.8843
	*tanh*	0.9219	0.9260	0.8842
8	*linear*	0.9206	0.9257	0.8830
	*minmax*	0.9238	0.9311	0.8838
	*log_minmax*	0.9207	0.9283	0.8844
	*tanh*	0.9161	0.9143	0.8836
7	*linear*	0.9177	0.3989	0.8850
	*minmax*	0.9194	0.9250	0.8841
	*log_minmax*	0.9218	0.9236	0.8833
	*tanh*	0.9131	0.9033	0.8851
6	*linear*	0.8952	0.9008	0.8871
	*minmax*	0.9144	0.8839	0.8842
	*log_minmax*	0.9111	0.9076	0.8844
	*tanh*	0.8702	0.8782	0.8788
5	*linear*	0.3722	0.8442	0.8802
	*minmax*	0.9031	0.9058	0.8810
	*log_minmax*	0.3948	0.8878	0.8812
	*tanh*	0.8247	0.3306	0.8670
4	*linear*	0.8500	0.2745	0.8587
	*minmax*	0.8678	0.8702	0.8775
	*log_minmax*	0.8649	0.3848	0.8734
	*tanh*	0.7491	0.8464	0.3017
3	*linear*	0.7928	0.8135	0.8190
	*minmax*	0.3391	0.7900	0.8530
	*log_minmax*	0.7664	0.8023	0.8564
	*tanh*	0.6922	0.2833	0.7985
2	*linear*	0.3006	0.6700	0.7065
	*minmax*	0.7371	0.3391	0.3466
	*log_minmax*	0.7908	0.7369	0.3110
	*tanh*	0.7216	0.7549	0.2309
1	*linear*	0.6700	0.3300	0.3300
	*minmax*	0.6706	0.7003	0.6717
	*log_minmax*	0.7171	0.7459	0.2121
	*tanh*	0.7171	0.7459	0.2121

## Supplementary Materials

The software of presented anomaly detection system is available online at https://bitbucket.org/maciekwielgosz/anomaly_detection.

## Abbreviations

The following abbreviations are used in this manuscript:

ADC	Analog-to-Digital Converter
ASIC	Application-Specific Integrated Circuit
AUC	Area Under Curve
CALS	CERN Accelerator Logging Service
CERN	European Organization for Nuclear Research
EP	Electronics for Protection Section
FPGA	Field-Programmable Gate Array
GRU	Gated Recurrent Unit
IF	Isolation Forest
LHC	Large Hadron Collider
LSTM	Long Short-Term Memory
MCD	Minimum Covariance Determinant
MPE	Machine Protection and Electrical Integrity Group
NN	Neural Network
OC-SVM	One-Class Support Vector Machine
PM	Post Mortem
QPS	Quench Protection System
RBF	Radial Basis Function
RMSE	Root-Mean-Square Error
RNN	Recurrent Neural Network
ROC	Receiver Operating Characteristic
TE	Technology Department

## Appendix A. Data Quantization

This section presents the equations that can help to better understand the data quantization performed during experiments.

### Appendix A.1. Adaptive Data Quantization

*Adaptive* data quantization principle of operation is mapping the input space to a fixed number of categories (bins) in such a way, that all categories have (ideally) the same samples cardinality as described by Equations (A1) and (A3) (see Table A1 for notation).

(A1) $$S_{\mathrm{norm}}\Pi_{\mathrm{qa}}\left(m\right)S_{\mathrm{qa}}:{\left\{0\ldots m-1\right\}}^{1\times n},$$

(A2) $$\Pi_{\mathrm{qa}}\left(m\right):\underset{x\in S_{\mathrm{norm}}}{⋀}\underset{y\in S_{\mathrm{qa}}}{⋁}y=\left\{\begin{array}{c} \;\begin{array}{c} {\mathrm{edges}}_{y}⩽x\;\cdot \;m<{\mathrm{edges}}_{y+1} \end{array}\\ \;\mathrm{if}\;x<1\\ y=m-1\;\mathrm{if}\;x=1, \end{array}\right.$$

(A3) $$\mathrm{edges}:\underset{0⩽y⩽m}{⋀}{\mathrm{edges}}_{y}=\left\{\begin{array}{c} 0\;\mathrm{if}\;y=0\\ \;\begin{array}{c} \mathrm{srt}\_{\mathrm{samples}}_{y\;\cdot \;\left\lceil \frac{n}{m}\right\rceil } \end{array}\\ \;\mathrm{if}\;0<y<m\\ 1\;\mathrm{if}\;y=m. \end{array}\right.$$

### Appendix A.2. Cumulative Amplitude Data Quantization

*Cumulative_amplitude* method is based on the idea of equalizing the sum of the samples amplitudes in each bin. As in *adaptive* algorithm, before edges selection, the samples are normalized and sorted. Then, the threshold value $\Theta_{m}$ is computed as a sum of amplitudes of samples left in the dataset divided by the required edges number (A4).

(A4) $$\Theta_{m}=\frac{\sum \mathrm{srt}\_\mathrm{samples}}{m},$$

(A5) $$\mathrm{edges}:\underset{0⩽y⩽m}{⋀}{\mathrm{edges}}_{y}=\left\{\begin{array}{c} 0\;\mathrm{if}\;y=0\\ \;\begin{array}{c} \mathrm{srt}\_{\mathrm{samples}}_{\mathrm{idx}(y)} \end{array}\\ \;\mathrm{if}\;0<y<m\\ 1\;\mathrm{if}\;y=m, \end{array}\right.$$

(A6) $$\mathrm{idx}\left(y\right)=\left\{\begin{array}{c} -1\;\mathrm{if}\;y=0\\ \;\min\left(k\right):\left(\sum_{i=\mathrm{idx}(y-1)+1}^{k}\left|\mathrm{srt}\_{\mathrm{samples}}_{i}\right|\right)⩾\Theta_{m}\\ \mathrm{if}\;0<y<m. \end{array}\right.$$

Contrary to the *adaptive* approach of determining the edges based on the samples count, in *cumulative_amplitude* the edge value is chosen when the sum of samples’ amplitudes crosses this threshold (A5)–(A6).

**Table A1:** Notation used in quantizaton Equations (A1)–(A6).

Symbol	Meaning
*n*	number of samples
*m*	number of classes (categories, bins); $m\in N_{>0}$
$S_{\mathrm{norm}}$	normalized input space
$S_{\mathrm{qa}}$	signal space after *adaptive* quantization
${\mathrm{edges}}_{i}$	*i*-th quantization edge, see (A3)
$\mathrm{srt}\_{\mathrm{samples}}_{i}$	*i*-th sample in the ascending sorted array of all available signal samples
